# In Patients with Cardiogenic Shock, Extracorporeal Membrane Oxygenation Is Associated with Very High All-Cause Inpatient Mortality Rate

**DOI:** 10.3390/jcm13123607

**Published:** 2024-06-20

**Authors:** Mohammad Reza Movahed, Arman Soltani Moghadam, Mehrtash Hashemzadeh

**Affiliations:** 1 College of Medicine, University of Arizona Sarver Heart Center, 1501 North Campbell Avenue, Tucson, AZ 85724, USA; 2 College of Medicine, University of Arizona, Phoenix, AZ 85004, USA

**Keywords:** MCS, mechanical circulatory support, ECMO/IABP/Tandem/Impella, CS shock, cardiogenic, shock, cardiac assist device, artificial heart, IABP, Impella, cardiac arrest

## Abstract

**Background:** The goal of this study was to evaluate the effect of extracorporeal membrane oxygenation (ECMO) on mortality in patients with cardiogenic shock excluding Impella and IABP use. **Method:** The large Nationwide Inpatient Sample (NIS) database was utilized to study any association between the use of ECMO in adults over the age of 18 and mortality and complications with a diagnosis of cardiogenic shocks. **Results:** ICD-10 codes for ECMO and cardiogenic shock for the available years 2016–2020 were utilized. A total of 796,585 (age 66.5 ± 14.4) patients had a diagnosis of cardiogenic shock excluding Impella. Of these patients, 13,160 (age 53.7 ± 15.4) were treated with ECMO without IABP use. Total inpatient mortality without any device was 32.7%. It was 47.9% with ECMO. In a multivariate analysis adjusting for 47 variables such as age, gender, race, lactic acidosis, three-vessel intervention, left main myocardial infarction, cardiomyopathy, systolic heart failure, acute ST-elevation myocardial infarction, peripheral vascular disease, chronic renal disease, etc., ECMO utilization remained highly associated with mortality (OR: 1.78, CI: 1.6–1.9, *p* < 0.001). Evaluating teaching hospitals only revealed similar findings. Major complications were also high in the ECMO cohort. **Conclusions:** In patients with cardiogenic shock, the use of ECMO was associated with the high in-hospital mortality regardless of comorbid condition, high-risk futures, or type of hospital.

## 1. Introduction

Cardiogenic shock, a devastating clinical consequence of acute myocardial infarction (AMI) and the primary contributor to post-MI mortality, occurs in approximately 5–15% of patients experiencing AMI due to deleterious effects of severe ischemia on left ventricular function [1,2]. It is characterized as systolic blood pressure below 90 mm Hg for 30 min or using inotropic agents to keep systolic blood pressure over 90 mm Hg and existing end-organ damage with subsequent diminished tissue perfusion and increased cardiac filling pressures [3]. Managing such conditions may sometimes require mechanical circulatory support (MCS) such as ECMO (extracorporeal membrane oxygenation), IABP (intra-aortic balloon pump), and Impella (an axial flow pump) to ensure hemodynamic support and boost myocardial function as a temporary bridging option [4]. Nevertheless, consensus is lacking regarding the beneficial effects of ECMO on mortality following cardiogenic shock, with inconclusive data available.

Currently, trends in the use of MCDs in the last ten years in the USA show an upward trend in the use of Impella and ECMO [5]. The first trial comparing the efficacy of IABP with standard treatment (IABP-SHOCK II trial) demonstrated no significant difference in the rate of 30-day mortality post-MI complicated by cardiogenic shock [6]. Furthermore, in a recent 6-year follow-up study conducted on this trial, there were no statistically significant differences observed in terms of mortality, recurrent myocardial infarction, stroke, repeat revascularization, or rehospitalization for cardiac reasons between the groups [7]. Interestingly, as recent data have shown the efficacy of IABP use in 30-day and 1-year mortality reduction following cardiogenic shock, given the incongruity within the data and the importance of the subsequent analysis in IABP Shock II follow-up, several guidelines have adjusted the recommended class for the use of IABPs in cardiogenic shock in ACS patients to a lower category. The European Society of Cardiology (ESC) and European Association for Cardio-Thoracic Surgery (EACTS) guidelines have revised the recommended classification, moving from a Class I to a Class III B recommendation (indicating not recommended for routine use in cardiogenic shock due to ACS). Similarly, the American College of Cardiology Foundation (ACCF) and the American Heart Association (AHA) guidelines have downgraded it to a Class IIb B recommendation. (weak usefulness or unknown/unclear/uncertain) [8,9,10,11]. While a recent meta-analysis, consisting of data from more than 10,000 patients, has shown improved mortality reduction outcomes with IABPs over ECMO and Impella, no reasonable grounds seem to exist for explaining this reclassification by guidelines [12]. We recently presented our data comparing Impella and intra-aortic balloon pumps (IABPs) in patients with cardiogenic shock, revealing the highest mortality with the use of Impella and the lowest mortality with IABPs [13]. 

VA-ECMO (veno-arterial ECMO) is another option for treating cardiogenic shock in patients following AMI. VA-ECMO employs a centrifugal flow pump, membrane oxygenator, and cannulas for venous inflow and arterial outflow [4,13]. Additional ports may be utilized for ultrafiltration and hemodialysis. Deoxygenated blood from a central vein passes through the membrane oxygenator, where the pCO_2_, pO_2_, and pH are adjusted, before being reintroduced into systemic circulation via the pump. Cardiac support can reach up to 6–7 L/min [14,15]. Cannulation options include central placement in the right atrium and ascending aorta for physiological circulation, primarily for post-cardiotomy patients, and peripheral approaches such as femoral–femoral or upper extremity arteries, allowing for retrograde or anterograde perfusion [16,17]. Continuous monitoring of hemodynamics and blood gases is crucial after ECMO initiation, with targets for flow, mean arterial pressure, and oxygen saturation aimed to promote myocardial recovery and organ function [18,19].

In a recent meta-analysis, VA-ECMO demonstrated no decrease in the 30-day mortality rate when compared to medical therapy in individuals experiencing infarct-related cardiogenic shock. Moreover, there was an observed rise in instances of major bleeding and vascular complications [20]. This meta-analysis only included 567 patients, which does not seem to add ample evidence to the literature. While data from NIS revealed an uptrend in the use of ECMO and a downtrend in the use of IABPs in patients with cardiogenic shock and ACS, there remains uncertainty regarding the efficacy of ECMO and its comparison to IABP use in mortality reduction [21]. 

A thorough examination of a large cohort is imperative to establish more conclusive evidence regarding the comparative mortality outcomes associated with the use of ECMO. Utilizing a retrospective analysis of data from the Nationwide Inpatient Sample (NIS) database among adult patients, we sought to achieve the largest possible sample size for a study of this nature. 

## 2. Methods

### 2.1. Data Source

The dataset employed in this research, known as the Nationwide Inpatient Sample (NIS), was formulated by the Agency for Healthcare Research and Quality (AHRQ) as a component of the Healthcare Cost and Utilization Project (HCUP). This database is de-identified, falls under the exemption from Institutional Review Board (IRB) approval, and is accessible to researchers and policymakers for the examination of nationwide patterns in healthcare utilization and results. The NIS encompasses details on both primary and secondary diagnoses and procedures, discharge vital status, and demographic information from almost one-fifth of all community hospitals in the United States.

### 2.2. Sample Selection

This retrospective analysis included individuals aged 18 and above who were discharged from a Nationwide Inpatient Sample (NIS) hospital between 2016 and 2020. The inclusion criterion involved a specific International Classification of Diseases, Tenth Revision, and Clinical Modification (ICD-10-CM) code: Cardiogenic Shock (R57.0). To mitigate the impact of confounding variables, a multivariate analysis was conducted, adjusting for 47 factors, including age, gender, race, and various comorbidities. The considered comorbidities encompassed conditions such as smoking, diabetes mellitus (250), chronic kidney disease, peripheral vascular diseases, cardiomyopathy, systolic heart failure, three-vessel PCI, left main STEMI, STEMI, anterior wall STEMI, cachexia, morbid obesity, obesity, chronic liver disease, atrial fibrillation/flutter, COPD, all valvular heart disease, history of stroke, acute lactic acidosis, cardiac arrest, mechanical ventilation, renal replacement therapy, heart failure, presence of aortocoronary bypass graft, right ventricular infarction, and rotational atherectomy (see Table 1). Comorbidities exhibiting significant *p*-values were included in the multivariate analysis for further adjustment. Additionally, we scrutinized outcome data, making comparisons between teaching hospitals and rural facilities.

### 2.3. Statistical Analysis

Patient demographic, clinical, and hospital characteristics are presented as percentages in the tables. Odds ratios and corresponding 95% confidence intervals were computed for continuous variables and proportions, while categorical variables have associated 95% confidence intervals. Temporal trends were evaluated using chi-squared analysis for categorical outcomes and univariate linear regression for continuous variables. Multivariable logistic regression was employed to determine the odds of binary clinical outcomes concerning patient and hospital characteristics, as well as the odds of clinical outcomes over time. All analyses incorporated population discharge weights. All *p*-values are two-sided, and a significance level of *p* < 0.05 was adopted. The analysis was conducted using STATA 17 (Stata Corporation, College Station, TX, USA). Cardiogenic shock occurrence and in-hospital mortality rates were computed annually to examine trends (2016–2020) and collectively for the final analysis.

## 3. Results

### 3.1. Mortality

**Figure 1:**
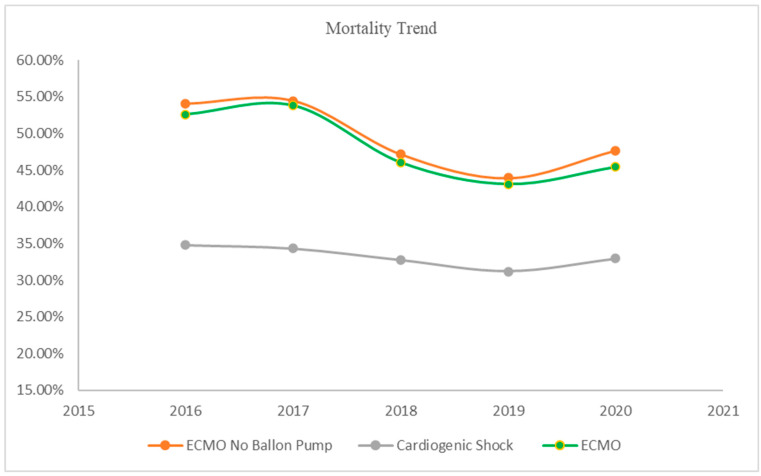
Mortality trends over years with ECMO.

In this retrospective analysis conducted from 2016 to 2020 using ICD-10 codes for ECMO, IABPs, and cardiogenic shock, a comprehensive examination of 796,585 patients (mean age 66.5 ± 14.4) with a diagnosis of cardiogenic shock, excluding Impella use, was conducted. Among this cohort, 13,160 patients (mean age 53.7 ± 15.4) were treated with ECMO alone. The complete demographics of the patients are available in Table 1. The overall inpatient mortality rate for patients without mechanical circulatory support devices was 32.7%. Notably, the mortality rate varied among the different treatment groups, with a high mortality rate of 47.9% for those treated with ECMO (Figure 1). 

### 3.2. Multivariate and Subgroup Analysis

A multivariate analysis was performed, adjusting for a robust set of 47 variables, including age, gender, race, lactic acidosis, three-vessel intervention, left main myocardial infarction, cardiomyopathy, systolic heart failure, acute ST-elevation myocardial infarction, peripheral vascular disease, and chronic renal disease. Upon adjustment, ECMO utilization remained significantly associated with the highest mortality (OR: 1.78, CI: 1.6–1.9, *p* < 0.001), underscoring the considerable impact of ECMO on patient outcomes (Table 2).

### 3.3. Complications

Beyond mortality outcomes, an analysis of major complications revealed a higher incidence in the ECMO cohort (Table 3) including increased pericardial effusion (OR = 2.19 (1.84–2.60), *p* < 0.001), cardiac tamponade (OR = 3.40 (2.80–4.13), *p* < 0.001), acute posthemorrhagic anemia (OR = 2.93 (2.65–3.23), *p* < 0.001), hemolytic anemia (OR = 6.92 (3.08–15.56), *p* < 0.001), disseminated intravascular coagulation (OR = 6.07 (5.13–7.19), *p* < 0.001), cardiac perforation (OR = 2.58 (1.94–3.44), *p* < 0.001), procedural bleeding (OR = 11.63 (6.60–20.49), *p* < 0.001), intraoperative cardiac functional disturbances (OR = 4.04 (2.66–6.14), *p* < 0.001), and acute postprocedural respiratory failure (OR = 1.23 (1.03–1.48), *p* = 0.02). We found that hemolytic anemia, procedural bleeding, disseminated intravascular coagulation, and septal or ventricular ruptures had the highest association with mortality in a multivariate model (Table 4).

## 4. Discussion

Our retrospective study found that ECMO was associated with the highest inpatient all-cause mortality in patients with cardiogenic shock (47.9%). Additionally, a mortality rate of 32.7% was observed when no device was used.

Various studies have reported different mortality rates of ECMO in patients with cardiogenic shock. Overall, the results have been virtually heterogeneous, and the in-hospital mortality rate ranged from 40% to 60% [22,23,24,25,26,27]. Recent clinical trials comparing the efficacy of VA-ECMO and optimal medical treatment demonstrated that using ECMO in patients with cardiogenic shock did not improve early and long-term mortality [28,29]. Additionally, a recent meta-analysis comparing the efficacy of ECMO to medical treatment revealed no mortality benefit from ECMO use in 30 days; however, long-term results appeared to be in favor of ECMO use [30]. Similarly, our study showed ECMO is associated with the highest mortality. ECMO patients likely had much worse underlying conditions that we could not capture in our database and adjust for. For example, patients requiring ECMO usually are very hypoxic and require not only mechanical support but also external oxygenation, making them much sicker than common patients with cardiogenic shock. Higher complications associated with the use of ECMO including bleeding, thromboembolic events, infections, and neurologic and vascular complications could also contribute to the higher mortality found in our study [31,32,33]. These complications emphasize the importance of considering both efficacy and safety profiles when evaluating mechanical circulatory support devices in the context of cardiogenic shock.

In our multivariate analysis adjusting for 47 variables, including age, gender, race, lactic acidosis, three-vessel intervention, left main myocardial infarction, cardiomyopathy, systolic heart failure, acute ST-elevation myocardial infarction, peripheral vascular disease, and chronic renal disease, ECMO use remained associated with the highest mortality (OR: 1.78, CI: 1.6–1.9, *p* < 0.001). This aligns with a recent meta-analysis of 10,985 patients concluding that IABP use outperforms both Impella and ECMO in improving mortality [12]. The contrast between our results and recent guideline adjustments by the European Society of Cardiology (ESC), European Association for Cardio-Thoracic Surgery (EACTS), American College of Cardiology Foundation (ACCF), and the American Heart Association (AHA) prompts questions about the rationale behind downgrading device use [8,9,34]. Our data underscore the need to reconsider recent guideline changes, given their substantial impact on clinical practice and patient outcomes. The difference between guidelines and our study highlights the need for continuous scrutiny and updates. This ensures that guidelines accurately reflect the latest evidence.

Other MCDs could also be used in the setting of cardiogenic shock. Previous data showed that in a cohort of patients with cardiogenic shock, the highest mortality was observed with the use of Impella (40.7%), while the lowest was with the use of IABPs (25.1%) compared to no device use (34.2%). However, a recent clinical trial comparing a microaxial flow pump (Impella CP) plus medical therapy to medical therapy alone showed that the microaxial flow pump reduced the risk of mortality in 180 days (HR = 0.74; 95% [CI], 0.55 to 0.99) but was associated with a higher risk of complications [35].

With recent advances in the use of MCDs, especially ECMO, the high mortality rate with the use of ECMO is still concerning. Although ECMO is a feasible option in patients with cardiogenic shock and rapidly deteriorating conditions, several factors could be considered in its use. First, it is imperative to recognize that ECMO serves solely as a bridging strategy to definitive treatment, and its implementation should not be expedited until all potential complications are thoroughly considered. Secondly, recent studies have proposed models for predicting outcomes and selecting patients for ECMO use. Prior to ECMO initiation, factors predictive of adverse outcomes include advanced age, female gender, and elevated body mass index, alongside indicators of heightened illness severity such as renal, hepatic, or neurological impairment, prolonged mechanical ventilation, increased lactate levels, and diminished prothrombin activity [36,37]. Notably, the PRECISE score has been able to predict in-hospital mortality with a sensitivity of 89% in patients with cardiogenic shock requiring VA-ECMO [38]. Ultimately, the consideration of these factors becomes paramount in the clinical decision-making process regarding the initiation of ECMO support. This offers a chance to identify patients who are most likely to derive significant advantages from such an intervention.

## 5. Conclusions

Our study showed ECMO is associated with very high mortality despite adjustment for 47 variables in patients with cardiogenic shock. It is very likely that ECMO patients had much worse underlying conditions that we could not capture in our database and adjust for. For example, patients requiring ECMO usually are very hypoxic, which requires not only mechanical support but also external oxygenation, making them much sicker than common patients with cardiogenic shock. Higher complications associated with the use of ECMO including bleeding, thromboembolic events, infections, and neurologic and vascular complications could also contribute to higher mortality found in our study. These findings underscore the critical importance of correctly selecting between different cardiac support devices in managing cardiogenic shock patients. Furthermore, we found that complication-related mortality is highest in patients suffering from disseminated intravascular coagulation, procedural bleeding, and septal or myocardial rupture. The highest risk was related to disseminated intravascular coagulation, suggesting that prolonged use of ECMO should be avoided as much as possible to reduce this risk and thus reduce mortality.

## 6. Limitations

First of all, the retrospective and non-randomized nature of our study might undermine the generalizability of our results. Although a multivariable adjustment was utilized for our analysis, there might have been unmeasured variables that were not taken into account. We employed ICD-10 coding, acknowledging its inherent limitations in providing precise diagnosis. Moreover, it is challenging to evaluate the rationale behind clinicians’ decision-making processes regarding the selection of ECMO or no device in each patient. In fact, it could not be ruled out that the patients receiving ECMO were sicker in nature. The complexities involved in understanding the factors influencing such choices, including patient-specific considerations and clinical judgment, remain beyond the scope of our current analysis.

## Figures and Tables

**Table 1:** Baseline characteristics of patients with cardiogenic shock.

	Cardiogenic Shock	No ECMO	ECMO	ECMO, No Balloon Pump	*p*-Value
Age N (Mean ± SD)	796,585 (66.57 ± 14.40)	779,225 (66.83 ± 14.28)	17,360 (54.87 ± 15.40)	13,160 (53.72 ± 15.43)	<0.001
Mortality	33.07%	32.74%	47.91%	49.05%	<0.001
Gender %					<0.001
Male	62.00%	61.91%	66.16%	64.29%	
Female	38.00%	38.09%	33.84%	35.71%	
Race %					<0.001
White	67.40%	67.46%	64.27%	63.34%	
Black	16.40%	16.39%	17.01%	17.80%	
Hipanic	8.99%	9.00%	8.74%	8.80%	
Asian/Pac Isl	3.32%	3.31%	3.70%	3.58%	
Native American	0.67%	0.67%	0.91%	1.07%	
Others	3.22%	3.17%	5.37%	5.41%	
Smoking	23.11%	23.30%	14.92%	15.05%	<0.001
Peripheral Vascular Diseases	5.27%	5.31%	3.08%	2.96%	<0.001
Cardiomyopathy	41.98%	42.17%	33.41%	29.86%	<0.001
Diabetes	39.48%	39.77%	26.56%	25.87%	<0.001
CKD	39.78%	40.15%	22.96%	21.39%	<0.001
Systolic Heart Failure	52.81%	52.93%	47.35%	43.84%	<0.001
Three-Vessel PCI	0.56%	0.55%	1.18%	0.80%	<0.001
Left main STEMI	0.12%	0.11%	0.52%	0.46%	0.56
STEMI	20.46%	20.42%	22.09%	18.73%	<0.001
Non-STEMI	18.35%	18.52%	10.92%	9.19%	<0.001
Anterior Wall STEMI	5.89%	5.82%	9.27%	7.56%	<0.001
History of MI	12.03%	12.17%	5.88%	5.32%	<0.001
Cachexia	2.62%	2.64%	1.84%	1.98%	0.005
Morbid Obesity	8.29%	8.27%	8.99%	9.80%	<0.001
Obesity	8.42%	8.42%	8.24%	8.13%	<0.001
Chronic Liver Disease	19.18%	18.74%	38.88%	39.06%	<0.001
Atrial Fibrillation/Flutter	43.54%	43.76%	33.87%	32.60%	<0.001
COPD	23.46%	23.73%	11.18%	11.06%	<0.001
All Valvular Heart Disease	22.73%	22.88%	20.54%	18.35%	<0.001
History of Stroke	2.02%	2.05%	0.86%	0.80%	0.006
Acute Lactic Acidosis	36.93%	36.68%	48.30%	48.44%	<0.001
Cardiac Arrest	9.57%	9.49%	13.10%	13.34%	<0.001
Mechanical Ventilation	45.96%	45.47%	68.23%	69.83%	<0.001
Renal Replacement Therapy	12.41%	12.11%	26.04%	26.75%	<0.001
Heart Failure	70.37%	70.44%	67.25%	64.67%	<0.001
Presence of Coronary Angioplasty and Graft	9.17%	9.22%	6.94%	6.00%	<0.001
Presence of Aortocoronary Bypass Graft	7.83%	7.91%	4.35%	4.48%	0.1
Presence of Cardiac Pacemaker	3.14%	3.18%	1.24%	1.03%	0.04
Prosthetic Heart Valve	2.72%	2.73%	2.42%	2.51%	<0.001
Presence of Automatic (Implantable) Cardiac Defibrillator	8.27%	8.37%	4.03%	3.76%	0.002
Coronary Angioplasty Status	1.04%	1.04%	0.86%	0.80%	0.24
Right Ventricular Infarction	1.35%	1.34%	1.73%	1.14%	<0.001
Rotational Atherectomy	0.10%	0.10%	0.14%	0.11%	0.09

**Table 2:** Mortality is very high with the use of ECMO regardless of any subgroup.

Mortality	ECMO, No Balloon Pump	Risk Ratio (CI)	*p*-Value
Age < 50	41.24%	2.73 (2.40–3.11)	<0.001
Age ≥ 50	53.48%	2.11 (2.00–2.22)	
Male	48.23%	2.12 (2.00–2.25)	<0.001
Female	50.53%	1.81 (1.68–1.95)	
Diabetes	52.72%	2.07 (1.91–2.24)	0.73
No Diabetes	47.77%	2.03 (1.92–2.15)	
STEMI	54.56%	1.95 (1.79–2.12)	0.009
No STEMI	47.78%	2.24 (2.11–2.37)	
Non-STEMI	50.83%	2.20 (1.92–2.51)	0.13
No Non-STEMI	48.87%	1.97 (1.87–2.07)	
Left main STEMI	66.67%	2.94 (1.73–5.02)	0.16
No Left main STEMI	48.97%	2.01 (1.92–2.10)	
Anterior Wall STEMI	51.76%	2.04 (1.76–2.35)	0.95
No Anterior Wall STEMI	48.83%	2.03 (1.93–2.13)	
Three-Vessel PCI	47.62%	1.92 (1.19–3.10)	0.84
No Three-Vessel PCI	49.06%	2.01 (1.92–2.11)	
Cardiac Arrest	56.13%	1.15 (1.04–1.28)	<0.001
No Cardiac Arrest	47.96%	2.17 (2.07–2.29)	
Acute Lactic Acidosis	56.47%	1.44 (1.36–1.53)	<0.001
No Acute Lactic Acidosis	42.08%	2.37 (2.21–2.55)	
Peripheral Vascular Diseases	57.69%	1.95 (1.58–2.41)	0.74
No Peripheral Vascular Diseases	48.79%	2.03 (1.93–2.12)	
Obesity	52.34%	2.59 (2.22–3.01)	<0.001
No Obesity	48.76%	1.96 (1.87–2.06)	
Smoking	50.76%	2.13 (1.91–2.38)	0.24
No Smoking	48.75%	1.99 (1.89–2.09)	
Hypertension	50.51%	2.12 (2.00–2.25)	<0.001
No Hypertension	47.22%	1.80 (1.66–1.94)	

**Table 3:** Complications in patients with ECMO vs. IABP.

Complications	Balloon Pump, No ECMO	ECMO, No Balloon Pump	*p*-Value	Odds Ratio (CI)
Pericardial effusion	3.72%	7.79%	<0.001	2.19 (1.84–2.60)
Cardiac tamponade	1.90%	6.19%	<0.001	3.40 (2.80–4.13)
Postprocedural acute kidney failure	0.42%	0.34%	0.59	0.81 (0.39–1.71)
Acute posthemorrhagic anemia	28.90%	54.33%	<0.001	2.93 (2.65–3.23)
Acquired hemolytic anemia	0.07%	0.49%	<0.001	6.92 (3.08–15.56)
Postprocedural hemorrhage	0.96%	5.09%	<0.001	5.55 (4.42–6.97)
Acute postprocedural respiratory failure	4.49%	5.47%	0.02	1.23 (1.03–1.48)
Disseminated intravascular coagulation	1.58%	8.89%	<0.001	6.07 (5.13–7.19)
Cardiac perforation (accidental puncture and laceration of a circulatory system organ)	0.87%	2.20%	<0.001	2.58 (1.94–3.44)
Procedural bleeding	0.10%	1.18%	<0.001	11.63 (6.60–20.49)
Intraoperative cardiac functional disturbances	0.33%	1.33%	<0.001	4.04 (2.66–6.14)
Postprocedural cerebrovascular infarction	0.07%	0.15%	0.18	2.12 (0.70–6.45)
Amputation of limb	0.06%	0.15%	0.09	2.70 (0.86–8.49)
Hemopericardium as current complication following acute myocardial infarction	0.11%	0.04%	0.29	0.34 (0.05–2.49)
Ventricular septal defect as current complication following acute myocardial infarction	0.65%	0.61%	0.82	0.94 (0.56–1.59)
Rupture of cardiac wall without hemopericardium as current complication following acute myocardial infarction	0.15%	0.11%	0.66	0.77 (0.23–2.52)
Rupture of chordae tendineae as current complication following acute myocardial infarction	0.32%	0.46%	0.27	1.42 (0.76–2.63)
Other current complications following acute myocardial infarction	0.09%	0.08%	0.8	0.82 (0.19–3.56)

**Table 4:** Mortality risk based on the occurrence of complications in the adjusted model.

	*p*-Value	Odds Ratio	95% CI for OR	
			Lower	Upper
Pericardial effusion	0.12	0.8	0.61	1.06
Cardiac tamponade	0.38	1.14	0.85	1.52
Postprocedural acute kidney failure	0.97	0.98	0.32	3.02
Acute posthemorrhagic anemia	<0.001	**0.73**	**0.63**	**0.84**
Acquired hemolytic anemia	0.26	1.79	0.65	4.97
Postprocedural hemorrhage	0.013	1.45	1.08	1.96
Acute postprocedural respiratory failure	0.45	1.12	0.83	1.5
Disseminated intravascular coagulation	<0.001	3.06	2.32	4.03
Cardiac perforation (accidental puncture and laceration of a circulatory system organ)	0.21	1.36	0.84	2.19
Procedural bleeding	0.15	1.68	0.84	3.36
Intraoperative cardiac functional disturbances	0.71	1.12	0.61	2.08
Postprocedural cerebrovascular infarction	0.45	0.37	0.03	4.76
Amputation of limb	0.44	0.41	0.04	4.02
Hemopericardium as current complication following acute myocardial infarction	NA	1		
Ventricular septal defect as current complication following acute myocardial infarction	0.003	2.89	1.45	5.78
Rupture of cardiac wall without hemopericardium as current complication following acute myocardial infarction	0.35	2.86	0.32	25.53
Rupture of chordae tendineae as current complication following acute myocardial infarction	0.39	0.68	0.28	1.63
Other current complications following acute myocardial infarction	0.81	1.42	0.09	22.91

## Data Availability

NIS data base publically available.
